# What to Do about Childhood Obesity?

**DOI:** 10.3390/ijerph16203902

**Published:** 2019-10-15

**Authors:** Roohi Kharofa, Robert Siegel, Kristin Stackpole

**Affiliations:** 1 Cincinnati Children’s Hospital, Cincinnati, OH 45229, USA; Roohi.Kharofa@cchmc.org (R.K.); Kristin.Stackpole@cchmc.org (K.S.); 2 College of Medicine, University of Cincinnati, Cincinnati, OH 45229, USA

Non-communicable diseases led by cardiovascular disease are all, in part, consequences of obesity and are now the number one cause of mortality worldwide [1]. According to the World Health Organization, obesity rates have nearly tripled since 1975 and over 40% of the world’s adults are overweight or obese [2,3]. The roots of this health crisis are established in early childhood and thus understanding the consequences of childhood obesity and developing strategies to prevent and treat childhood obesity are of the utmost importance. With this special issue of *IJERPH*, we feature 20 articles that address nutritional and activity interventions as well as other treatment issues that relate to the critically important topic of childhood obesity.

Several of the articles in this issue are dedicated to the nutritional aspects of childhood obesity. Low carbohydrate or ketogenic dieting has become increasing popular, but there is limited evaluation in adolescents. Pratt, Eneli et al. in two articles describe the results and acceptance of a low carbohydrate dietary intervention with calories limited to 1200–1800 per day [4,5]. During their 12-month intervention, the authors found that adherence dropped from 50% during the first three months to less than 15% at 6 months. While a peak, mean weight loss of 5.5 kg was described at 3 months, mean weight loss was 1.3 kg at 12 months somewhat similar to results in other pediatric studies of low carbohydrate diets [6,7]. Fahenkamp et al. further our understanding of eating behavior by studying whether experimental avoidance (EA) may be a mechanism in mediating the association of food cravings and problematic eating behavior. Interestingly, they found that EA mediated the association between food cravings and both emotional eating and cognitive restraint, but not uncontrolled eating [8]. Schaller et al. report their experience in treating youth with severe obesity who are challenged with complex social issues and family dynamics [9]. The authors describe the need for the integration of medical tertiary care, mental health services and community resources with these challenging patients and their experience with anti-obesity medications and meal replacements. Lehto et al. describe a comprehensive survey of 864 children in 66 Finnish preschools [10]. They measured several key components of energy balance including food intake by three-day food records, food frequency questionnaires, physical activity by 7-day accelerometer data, a sedentary behavior log and stress levels by hair cortisol sample. They found that low parental educational level correlated was a risk factor for high sugar-sweetened beverage intake, more screen time and lower consumption of fruits and vegetables. Gajewska et al. explored the effect of childhood obesity and iron status. They compared multiple biomarkers of iron status including serum ferroportin, hepcidin, ferritin, and iron concentration in 40 children with obesity and 40 children with normal weight status [11]. Their results show that in children with obesity and normal iron intake, the mean ferritin/hepcidin and ferroportin/hepcidin ratios were lower than controls without biochemical signs of iron deficiency. Not only do interventions have to be effective, if we are going to address obesity on a population level, interventions have to be practical and cost-effective. Zanganeh and colleagues in this issue present and extensive review of cost-effective with an economic evaluation of obesity interventions [12]. Out of 35 of 39 were cost-effective. The majority of “behavioural and policy” preventions were cost-effective and even cost-saving.

The exercise-focused articles in this special issue were diverse, ranging from research focusing on how to identify children at high risk of poor cardiac fitness to studies supporting the positive impacts of physical activity, both metabolically and emotionally. Milne et al. worked toward the validation of a cardiorespiratory fitness measure, entitled the Modified-Shuttle-Test-Paeds (MSTP) [13]. The authors identified a very strong predictive relationship between the more practical MSTP and the gold standard measurement of VO2peak. Given the associated ease of use, the MSTP will enable mass measurement of students in the school setting as well as risk stratification at an individual level, allowing for targeted programming that could decrease a child’s likelihood of obesity and associated comorbidities. Tabacchi et al. also sought to identify high-risk children, albeit through a very different methodology [14]. They performed a latent class analysis to create fitness-related risk profiles, with each adolescents’ sex, age, parents’ weight status, parents’ education, and school type serving as significant predictors of class. Future use of this class analysis will not only allow for individual risk stratification but also for more broad city/school categorization, potentially setting the stage for public health interventions in early childhood. Moving from identification of at-risk youth to education, two studies looked at methodologies for increasing physical activity. Xiao Lau et al. created a physical activity module for adolescents based on focus groups with teens [15]. The teens identified key components of both content and presentation that could positively impact information uptake. Interestingly, adolescents preferred information presented in images (cartoon-based, colorful) or diagrams/mind maps. Patrick Lau et al. also looked at increasing physical activity in adolescents but instead utilized SMS messaging reminders at different frequencies and timings as their medium [16]. While the authors did not identify any significant differences in adolescents’ PA among the five groups (4 intervention, 1 control), they highlight the importance of studies looking at modalities to reach adolescents that are both contemporary and evidence-based. The remaining two exercise-related studies focused on presenting data on the positive outcomes of physical activity in youth. Zguira et al. set out to address the conflicting data on the impact of physical activity on glucose and LDL measurements [17]. They found that their eight-week individualized training program decreased glucose, TG, TC, LDL and leptin in obese adolescents with and without metabolic syndrome. Ievers-Landis et al. also found positive effects of physical activity [18]. Adolescents who were more confident in their ability to successfully engage in physical activity were less likely to report being teased about their weight, regardless of their BMI. The authors note that this finding has the potential for significant impact, given that focusing on exercise abilities would address weight, physical abilities, and emotional well-being simultaneously.

Finding effective strategies for the prevention and treatment of obesity in children and adolescents is essential. Several articles in this issue address new approaches or tools in weight management treatment, prevention, and research. Moore et al. evaluated a patient decision-aid promoting shared decision-making to be used in adolescents with severe obesity participating in weight management programs [19]. This aid described the risks, benefits, and indications of intensive lifestyle management alone versus bariatric surgery with lifestyle intervention. Lanzarote-Fernández and colleagues adapted and validated a Spanish version of the Family Health Behavior Scale [20]. This instrument could potentially not only assess family health habits but allow for comparison of data between different countries. An article by Bartelink et al. describes the use of a contextual action-oriented research approach (CARA) to study the implementation of a Dutch initiative, “The Healthy Primary School of the Future” [21]. The authors’ approach may serve as a model for further research efforts in health promotion in complex systems. Rodriguez-Ventura et al. studied a pilot program focusing on healthy lifestyle which considered the socio-cultural context of the participants and their parents [22]. The program involved group sessions and active parent participation. The study evaluated changes in anthropometric measurements in both children and parents. Also focusing on families, Frate et al. demonstrated that children as young as preschool already show behaviors that deviate from recommendations regarding screen and sedentary time, physical activity, and dietary intake arguing that obesity prevention strategies should be implemented on the familial level [23]. The argument to educate adolescents on healthy nutrition and weight management behaviors was made by Deierlein et al. [24]. They analyzed survey questions regarding weight perception and the desire to change weight in 10 to 15-year-olds. The performance of five-year-old children in the area of motor, perceptual, and social-emotional skills was analyzed based on BMI and gender by Madrona et al. [25]. Differences were found both in gender and BMI.

With this special issue of *IJERPH*, the authors collectively address many of the key areas that relate to the prevention, identification, and treatment of childhood obesity. Not only does their collective work expand our knowledge, but it also demonstrates how quickly and effectively we are addressing this important healthcare problem.
